# Deubiquitylation of hepatitis B virus X protein (HBx) by ubiquitin-specific peptidase 15 (USP15) increases HBx stability and its transactivation activity

**DOI:** 10.1038/srep40246

**Published:** 2017-01-11

**Authors:** Zhi-Jun Su, Jia-Shou Cao, Yan-Fang Wu, Wan-Nan Chen, Xinjian Lin, Yun-Li Wu, Xu Lin

**Affiliations:** 1 Key Laboratory of Ministry of Education for Gastrointestinal Cancer, Fujian Medical University, Fuzhou, China; 2 Department of Infectious Diseases, The First Hospital of Quanzhou Affiliated to Fujian Medical University, Quanzhou, China; 3 Fujian Key Laboratory of Tumor Microbiology, Fujian Medical University, Fuzhou, China

## Abstract

Hepatitis B virus X protein (HBx) plays important roles in viral replication and the development of hepatocellular carcinoma. HBx is a rapid turnover protein and ubiquitin-proteasome pathway has been suggested to influence HBx stability as treatment with proteasome inhibitors increases the levels of HBx protein and causes accumulation of the polyubiquitinated forms of HBx. Deubiquitinases (DUBs) are known to act by removing ubiquitin moieties from proteins and thereby reverse their stability and/or activity. However, no information is available regarding the involvement of DUBs in regulation of ubiquitylation-dependent proteasomal degradation of HBx protein. This study identified the deubiquitylating enzyme USP15 as a critical regulator of HBx protein level. USP15 was found to directly interact with HBx via binding to the HBx region between amino acid residues 51 and 80. USP15 increased HBx protein levels in a dose-dependent manner and siRNA-mediated knockdown of endogenous USP15 reduced HBx protein levels. Increased HBx stability and steady-state level by USP15 were attributable to reduced HBx ubiquitination and proteasomal degradation. Importantly, the transcriptional transactivation function of HBx is enhanced by overexpression of USP15. These results suggest that USP15 plays an essential role in stabilizing HBx and subsequently affects the biological function of HBx.

Hepatocellular carcinoma (HCC) is a common malignancy and chronic hepatitis B virus (HBV) infection is one of the major risks of developing HCC, which accounts for more than 80% of HCC cases in high-incidence region^1^. The pathogenesis and carcinogenesis of HBV-associated HCC mainly involve continuous liver inflammation response to virus infection and the integration of HBV DNA into the host genome to induce direct mutagenesis of diverse cancer-related genes^2^. Among the 7 HBV viral proteins, hepatitis B virus X protein (HBx) appears to possess the most pathogenic potential as it can associate with many proteins to transactivate a variety of viral and cellular genes involved in gene transcription, intracellular signal transduction, genotoxic stress response, protein degradation, cell cycle control and apoptosis^3^. Comprehensive studies have suggested that HBx is a promiscuous transactivator and plays pivotal roles in HBV replication and hepatocarcinogenesis^4^,^5^. A high incidence of HCC was observed in transgenic mice expressing HBx protein^6^,^7^ and knockdown of HBx expression in Hep3B cells markedly reduced xenograft tumor growth^8^. Therefore, HBx expression level is crucial in the pathogenesis of HBV-related HCC.

Like other viral proteins, HBx expressed within the host cell is degraded by the proteasome pathway although the mechanisms regulating this process remain to be elucidated^9^. 26 S proteasome complex^9^, heat shock protein 40 (Hsp40)^10^, HBV core proteins^11^, tumor suppressor p53^12^, transcriptional factor Id-1^13^, MDM2^14^ and Siah-1^15^ have been shown to contribute to destabilizing HBx protein in a proteasome-dependent manner. For instances, Id-1 destabilized HBx protein by facilitating the interaction between ubiquitinated HBx and proteasome subunit C8 while Id-1 itself had no influence on the ubiquitylation modification of HBx^13^. Tumor suppressor p53 is capable of increasing HBx ubiquitylation with an unknown mechanism^14^. A previous study demonstrated that HBx can be actively ubiquitinated with the host cell and undergo proteolysis, suggesting that HBx degradation is ubiquitin-dependent^9^. Removal of ubiquitin from mono- and poly-ubiquitylated proteins is also critical for rescuing them from degradative pathways or leading to the reversion of ubiquitin signaling. However, no deubiquitylating enzymes or deubiquitinases (DUBs) have been identified so far that might account for regulation of ubiquitylation-dependent proteasomal degradation of HBx protein. One DUB that has recently received a lot of attention, primarily through its well-documented association with various cancer-signaling pathways, is the ubiquitin-specific peptidase 15 (USP15). For example, USP15 was one of twelve DUBs identified from an siRNA screen that regulates the hepatocyte growth factor (HGF)-dependent cell scattering response in non-small cell lung cancer and pancreatic cancer cells^16^. A number of specific USP15 substrates have also been described, including the human papilloma virus (HPV) E6 oncoprotein^17^, the RING-box protein Rbx1^18^, the adenomatous polyposis coli (APC) tumour suppressor^19^, and the NF-κB inhibitor IκBα^20^.

Many of the HBx binding partners have been identified using immunoprecipitation or using classical yeast two-hybrid (Y2H) screenings. While some of these interactions with HBx have been validated, the physiologic relevance of such interactions remains largely uncertain. In an effort to identify HBx interacting proteins, we previously employed the CytoTrap yeast two hybrid (Y2H) system to screen for cellular proteins that may interact with HBx and have identified several new candidate proteins including USP15 (unpublished data). This study further explored and confirmed HBx as a novel USP15-binding partner and substrate. We found that USP15-mediated deubiquitylation protects HBx from proteasomal degradation thus increasing the stability and level of HBx protein as well as its transactivation activity. These results suggest that interventions directed at suppressing the level or functional activity of USP15 may be of therapeutic value in HBx-related hepatocarcinogenesis.

## Results

### HBx associates with USP15 *in vitro* and *in vivo*

**Figure 1:**
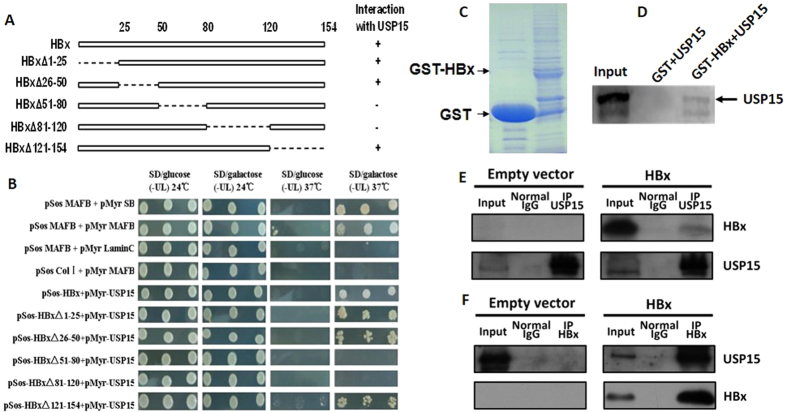
Interaction between HBx and USP15. (**A**) Schematic depiction of HBx and HBx mutants. The deleted region is indicated by a dashed line with amino acid position on both ends. The ability of each HBx variant to bind to USP15 was indicated as ( + ) or (−). (**B**) Analysis of interaction between HBx mutants and USP15 by CytoTrap two-hybrid assay. The paired plasmids were co-transformed into yeast strain cdc25Hα, three independent transformants were selected at 24 °C on SD/glucose(-UL) plates or SD/galactose (-UL) plates. Colonies were then patched onto SD/glucose(-UL) plates or SD/galactose(-UL) plates and grew at 37 °C. (**C**) Coomassie blue-stained SDS-PAGE gel showing bacterially expressed GST and GST-HBSP recombinant proteins. (**D**) Representative autoradiogram of in *vitro*-translated ^35^S-USP15 retained by GST-HBx fusion proteins from a GST pull-down assay. The input lane corresponds to the purified ^35^S-USP15 loaded with 1/10 the amount of ^35^S-labeled proteins used in the binding reactions. (**E,F**) Co-IP assay showing the interaction between HBx and endogenous USP15 in Huh7 cells transfected with empty vector of pcDNA3.1/myc-His(−) A or pHBx -FLAG. The immunoprecipitation of USP15 was detected for HBx and vice versa by Western blot analysis. The input served as expression and specificity control for the individual proteins.

To confirm the interaction of HBx with USP15 and map the domains of HBx involved in this interaction, HBx and a series of deletion mutants (Fig. 1A) were examined by CytoTrap two-hybrid assay for their ability to interact with USP15. Yeast transformant colonies harboring pMyr-USP15 with deletion mutant of HBxΔ51–80 or HBxΔ81–120 could not grow in galactose at 37 °C (Fig. 1B, rows 8 and 9), which indicates that the region between HBx amino acid residues 51 and 120 is required for the interaction with USP15. To further confirm the interaction between HBx and USP15 *in vitro*, the GST pull-down assay was performed. As shown in Fig. 1C and D, GST and GST-HBx protein were well expressed, and ^35^S-labeled USP15 was retained on the GST-HBx-conjugated sepharose whereas GST alone was not, indicating that USP15 could interact with HBx directly *in vitro*. To address a potential interaction between the two proteins in hepatocytes, we conducted an *in vivo* co-immunoprecipitation (Co-IP) study with the Huh7 cells transiently transfected with HBx expression vector pHBx-flag or empty vector. Figures 1E and F show that HBx was able to efficiently co-precipitate with endogenous USP15, and vice versa. Taken together, these results strongly suggested that HBx and USP15 interact specifically both *in vitro* and *in vivo*.

### HBx does not affect USP15 degradation or peptidase activities

**Figure 2:**
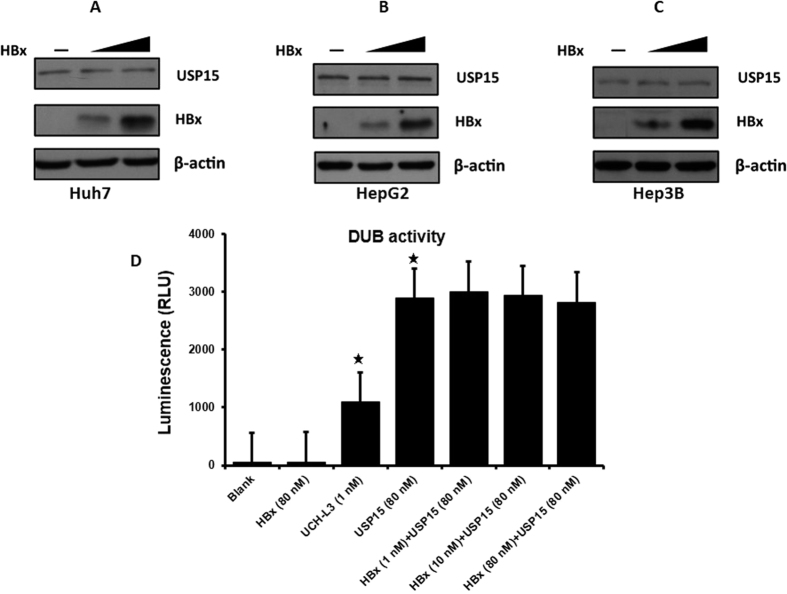
Effect of HBx on USP15 degradation or peptidase activities. (**A–C**) HBx had no effect on USP15 degradation. Endogenous USP15 protein level was analyzed in Huh7(A), HepG2 (B) and Hep3B (C) cell lines transfected with empty vector pcDNA3.1/myc-His(−) A or increasing amount of pHBx-FLAG, β-actin is included as a loading control. (**D**) HBx had no effect on USP15 peptidase activities. *In vitro* US15 peptidase activities was assessed by DUB-Glo Protease assay using a human recombinant USP15 with the addition of increasing amount of purified HBx. 1 nM of DUB protease UCH-L3 was used as a positive control. The luminescence was measured and was recorded as relative light units (RLU). Values were mean ± S.D of three separate experiments. *P < 0.05 vs blank control.

To assess the functional consequence of the interaction between USP15 and HBx, we first examined the effects of HBx on USP15 protein level and peptidase activities. As shown in Fig. 2A–C, the steady-state level of USP15 was not affected by HBx expression in Huh7, HepG2 and Hep3B cells. We also performed an *in vitro* USP15 peptidase activities by DUB-Glo Protease assay using a human recombinant USP15 with the addition of increasing amount of purified HBx. The results demonstrated that peptidase activities of USP15 was not affected by HBx (Fig. 2D). We therefore conclude that HBx does not impact USP15 protein level and its peptidase activity.

### USP15 affects HBx protein levels

**Figure 3:**
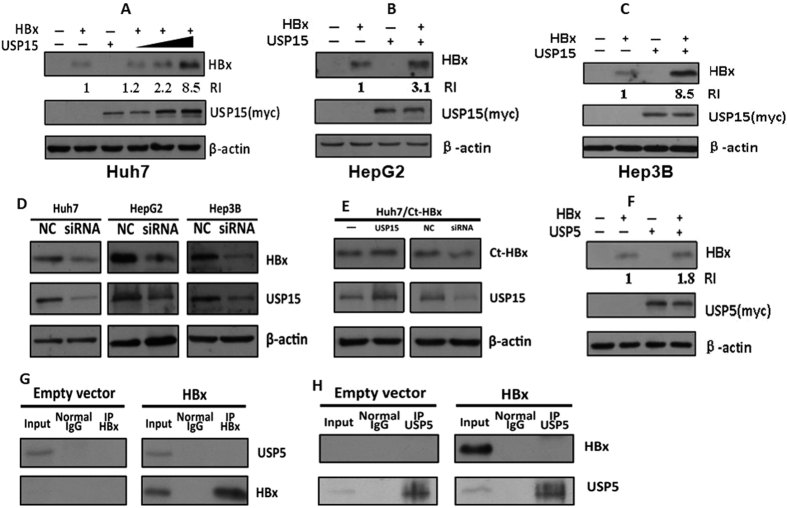
USP15 increases HBx protein levels. (**A–C**) USP15 co-expression increased HBx protein level. USP15 and HBx were detected by anti-myc or anti-FLAG antibody, respectively. β-actin is included as a loading control. Densitometry analysis of band signals was carried out using Image J software and the levels of HBx protein in cells transfected with pHBx-FLAG + pUSP15-myc were expressed as the relative intensity (RI) to that in the empty vector control pUSP5-myc or pHBx-FLAG after normalization to β-actin. A: Huh7cells co-transfected with 0.75 μg pHBx-FLAG and 2.25 μg pUSP15-myc; B: HepG2 cell lines co-transfected with 0.75 μg pHBx-FLAG and 2.25 μg pUSP15-myc; C: Hep3B cell lines co-transfected with 0.75 μg pHBx-FLAG and 2.25 μg pUSP15-myc. (**D**) Knocking down of endogenous USP15 decreased HBx protein level. Huh7, HepG2 and Hep3B cells were separately transfected with siRNA targeting USP15. HBx and USP15 were detected by anti-FLAG and anti-USP15 antibody. β-actin was included as a loading control. (**E**) Overexpression or knockdown of USP15 correspondingly increased or reduced HBx expression in Huh7/Ct-HBx cells that had been stably transfected with a carboxyl-terminal truncated form of HBx (Ct-HBx, depletion of amino acids 130–154). HBx and USP15 were detected by anti-FLAG and anti-USP15 antibody. β-actin was included as a loading control. (**F**) USP5 overexpression increased HBx protein level. Huh7 cells were co-transfected with 0.75 μg pHBx-FLAG and 2.15 μg pUSP5-myc (equal molar amount of 2.25 μg pUSP15-myc). HBx was analyzed by western blot, and relative intensity was calculated as mentioned above. (**G,H**) Co-IP assay of USP5 and HBx. Huh7 cells were transfected with empty vector pcDNA3.1/myc-His(−) A or pHBx -FLAG. The immunoprecipitation of HBx was detected for USP5 (**F**) and vice versa (**G**) by western blot analysis. The input served as expression and specificity control for the individual proteins.

We then tested whether USP15 could affect the HBx protein level. Huh7 cells were transfected with HBx alone or together with USP15. As shown in Fig. 3A, USP15 overexpression significantly increased HBx protein levels in a dose-dependent manner. A similar result was obtained in HepG2 and Hep3B cells (Fig. 3B and C). In contrast, knocking down endogenous USP15 by siRNA resulted in a significant reduction of HBx protein levels in Huh7, HepG2 and Hep3B cells (Fig. 3D). To examine whether over-expression or knockdown of USP15 affects the HBx protein levels in a HCC cell line with “endogenous” HBx we ectopically expressed a carboxyl-terminal truncated form of HBx (Ct-HBx, depletion of amino acids 130–154) in Huh7 cells (the stable cell line is designated here as Huh7/Ct-HBx). As shown in Fig. 3E, overexpression or knockdown of USP15 in the Huh7/Ct-HBx cells correspondingly increased or reduced Ct-HBx protein expression. To assess the specificity of USP15 in increasing HBx levels, we performed similar cotransfection experiments with another DUB, USP5 (also known as isopeptidase)^21^. We observed only a moderate increase of 1.8-fold in HBx protein levels in Huh7 cells expressing USP5 (Fig. 3F), as compared with 8.5-fold increase in USP15-expressing Huh7 cells. However, HBx and USP5 did not co-immunoprecipitate with each other in the cell extracts prepared from the co-transfected Huh7 cells (Fig. 3G,H), suggesting that the moderate increase in HBx levels by USP5 might be owing to the general ability of USP5 to disassemble polyubiquitin chains^22^ rather than the binding of USP5 to HBx.

### USP15 stabilizes HBx

**Figure 4:**
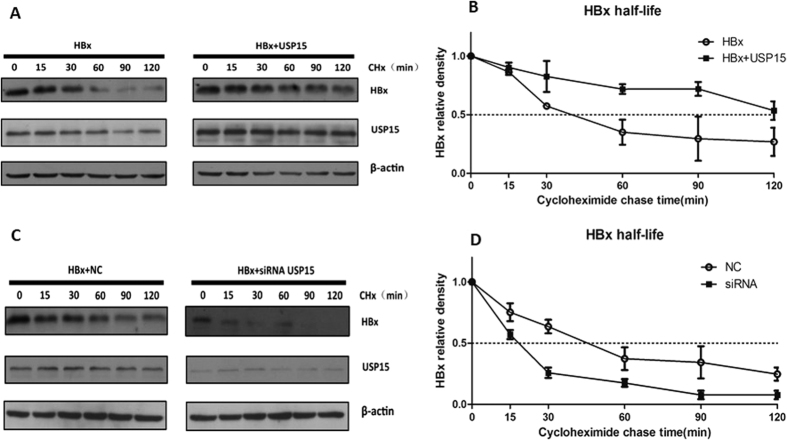
USP15 extends HBx half-life. (**A,B**) USP15 overexpression increased the HBx protein level by extending the half-life of HBx. Huh7 cells were transfected with pHBx-FLAG or in combination with pUSP15-myc. 48 h posttransfection, cells were treated with 100 μg/ml cycloheximide to inhibit *de novo* translation. Cells were lysed at the time points indicated, and the lysates were analyzed by western blotting with anti-FLAG or anti-USP15 antibody (**A**), and cumulative data from three experiments were shown (**B**). (**C,D**) USP15 suppression by RNA interference reduced HBx protein levels by decreasing the half-life of HBx. Huh7 cells were transfected with USP15 siRNA or negative control RNA. 48 h posttransfection, cells were treated with 100 μg/ml cycloheximide, and lysed at the time points indicated. The lysates were analyzed by western blotting (**C**), and cumulative data from three experiments were shown (**D**).

To determine whether the increased levels of HBx by co-expression of USP15 resulted from an extended half-life, we performed a cycloheximide chase experiment. Huh7 cells were transfected with HBx alone or in combination with USP15, then continually exposed to cycloheximide for different time periods up to 120 mins. Cell extracts were analyzed by Western blot with the specific antibodies. Figure 4A and B shows that USP15 expression led to a longer degradation time for the HBx-transfected cells, increasing HBx half-life from approximate 45 mins to 113 mins. To examine the effect of endogenous USP15 on the exogenously expressed HBx protein, Huh7 cells were co-transfected with HBx and USP15-targeting siRNA. As shown in Fig. 4C and D, knockdown of endogenous USP15 resulted in a significant decrease in the half-life of HBx from around 45 mins to 18 mins. There results clearly indicate that USP15 attenuates the degradation of HBx.

### USP15 attenuates HBx degradation through deubiquitination of HBx

**Figure 5:**
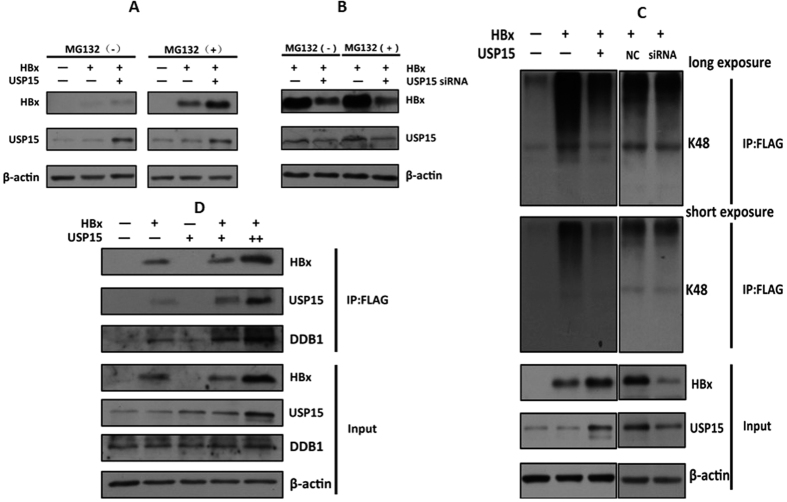
USP15 attenuates HBx degradation through deubiquitination of HBx. (**A,B**) Stabilization of HBx by USP15 through proteasomal pathway. Huh7 cells were transfected with pHBx-FLAG in combination with pUSP15-myc (**A**) or siRNA targeting USP15 (**B**). 20 h after transfection, cells were treated with 20 μM MG132 for 6 h, cell lysates were subjected to western blot using anti-FLAG antibody. (**C**) USP15 affected ubiquitination of HBx. Huh7 cells were transfected with pHBx-FLAG in combination with pUSP15-myc (left) or siRNA targeting USP15 (right). 20 h after transfection, cells were treated with 20 μM MG132 for 6 h. Total cell extracts were first subjected to immunoprecipitation using anti-FLAG antibody, and then immune complexes were assayed by western blot, and anti-K48 ubiquitin antibody was used to detect the ubiquitination levels of HBx. The input represented the expression level of HBx and USP15 in the transfected cells detected by anti-FLAG or anti-USP15 antibody. (**D**) USP15 did not compete with HBx to bind to E3 ubiquitin ligase complexes. Huh7 cells were transfected with pHBx-FLAG in combination with increasing amount of pUSP15-myc. Total cell extracts were first subjected to immunoprecipitation using anti-FLAG antibody, and then immune complexes were assayed by western blot for the presence of USP15 or DDBI. The input represented the expression level of HBx, USP15 and DDB1 in the transfected cells detected by anti-FLAG, anti-USP15 or anti-DDB1 antibody.

USP15 has been well documented to catalyze the deubiquitylation of most substrates^17^,^20^. Ubiquitination of HBx has been previously reported and both ubiquitin-dependent and -independent proteasomal degradation processes appears to be operative in its turnover^23^. To test if the effect of USP15 on HBx expression is proteasome-dependent, Huh7 cells were co-transfected with pHBx-FLAG and pUSP15-myc or USP15 targeting siRNA then treated with the proteasome inhibitor MG132. As shown in Fig. 5A, increasing HBx expression by ectopic expression of USP15 was further enhanced by treatment with the proteasome inhibitor MG132 whereas the reduced HBx protein levels resulting from knockdown of USP15 was rescued by treatment with the MG132 (Fig. 5B). Since a proteasome inhibitor can block the rapid breakdown of proteins by the ubiquitin pathway without affecting protein synthesis, it should cause a short-lived protein to accumulate in the cell if the protein is degraded through the ubiquitin-proteasome pathway. The observation that addition of MG132 led to higher HBx level at USP15 overexpression or rescued the reduction of HBx from USP15 knockdown indicates that the proteasome-dependent function of USP15 plays a primary role in regulation of HBx turnover. To confirm the deubiquitination of HBx by USP15, we performed an *in vivo* ubiquitination assay in which HBx was immunoprecipitated and detected by antibody against ubiquitin. Indeed, overexpression of USP15 diminished K48-linked polyubiquitination of HBx whereas inhibition of endogenous USP15 expression by siRNA-mediated depletion markedly increased K48-linked HBx polyubiquitination even though the protein level of HBx itself had been substantially reduced (Fig. 5C). In addition, for the reasons that E3 ubiquitin ligase has been shown to facilitate polyubiquitylation and proteasomal degradation of HBx^15^, we considered the possibility that USP15 may interfere with E3 ubiquitin ligase in its ability to target HBx for proteasomal degradation. As shown in Fig. 5D, despite the fact that USP15 dose-dependently increases HBx levels, it did not affect the expression levels of DDB1, a core component of E3 ubiquitin ligase complexes. Instead, with the increase of HBx levels, an association of USP15 with DDB1 was also increased. Therefore, these results indicate that USP15 protects HBx from proteasome-mediated degradation through reducing HBx ubiquitination rather than competing with HBx to bind to E3 ubiquitin ligase complexes.

**Figure 6:**
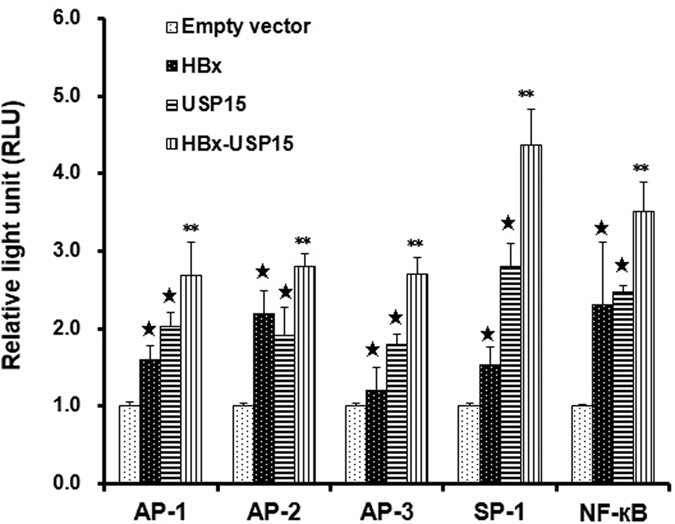
USP15 enhances the transactivation activity of HBx. Huh7 cells were co-transfected with pHBx-FLAG, and 0.4 μg each of cis-element luciferase reporter plasmid including pAP-1-luc, pAP-2-luc, pAP-3-luc, pSP-1-luc, and pNF-κB-luc. 48 h after transfection, cells were lysed and 30 μg protein were used for the detection of intracellular luciferase activity. The light intensity was measured and the relative luciferase unit (RLU) were obtained by comparison to that from the empty vector control pCDNA3.1/myc-His(−)A. Each transfection was performed in duplicate and repeated three times. *P < 0.05 vs empty vector, **P < 0.05 vs HBx or USP15.

USP15 enhances the transactivation activity of HBx. One of fundamental functions of DUBs is the specific deconjugation of ubiquitin from targeted proteins, which may rescue them from proteasome-dependent destruction and thus resume their original designated functions. Given the observations that USP15 binds to HBx, trims ubiquitin from HBx, and consequently increases HBx stability and protein level, we went on to explore whether the transcriptional transactivation function of HBx could be increased as a result of elevated HBx protein level due to the interaction between USP15 and HBx. As shown in Fig. 6, co-expression of USP15 enhanced the AP-1, AP-2, AP-3, SP-1 and NF-κB signal pathways transactivated by ectopically expressed HBx although these signal pathways were also affected to a lesser extent by USP15 overexpression alone (Fig. 6). This implied that the association of USP15 with HBx not only increases the stability of HBx but also augments HBx-mediated oncogenic signals.

## Discussion

HBx is an unstable protein having a short half-life, whose instability is considered to be attributed to rapid degradation through the ubiquitin-proteasome pathway^9^,^22^. Since ubiquitination is a dynamic and reversible process, and prolonged expression of the viral regulatory protein HBx is required for dysregulation of cell transcription and proliferation control, cells must possess a specific and also rapid deubiquitination machinery capable of such a tight control of HBx expression. However, mechanisms underlying HBx deubiquitination remains unknown. In our present study, we have identified the deubiquitylating enzyme USP15 as a key regulator of HBx protein levels. We demonstrate for the first time that USP15 specifically interacts with HBx and consequently increases HBx stability and the steady-state level by inhibition of HBx ubiquitination and degradation.

The physical and specific interaction between HBx and USP15 was confirmed from a series of comprehensive binding studies. To determine whether there is a possible association between HBx and USP15, perhaps under more physiologic condition, the CytoTrap yeast two-hybrid system was employed, and the results demonstrated that HBx could interact with USP15, and the region between HBx amino acid residues 51 and 80 is required for the interaction with USP15. Furthermore, by using the GST pull-down assay, we demonstrated that HBx was able to bind to USP15 in the absence of a cellular context. To assess the biological relevance of this interaction, HBx was transfected in Huh7 cells, and coimmunoprecipitation analyses showed that HBx interacted with endogenous USP15 and vice versa. These results clearly indicate that HBx and USP15 interact specifically both *in vitro* and *in vivo*.

USP15 was first cloned and characterized in 1999 and belongs to the largest ubiquitin specific protease (USP) group of deubiquitinating enzymes (DUBs)^23^. However, only recently some of its function and targets are being elucidated. USP15 has been reported to associate with COP9 signalosome (CSN), a multiprotein complex that regulates the ubiquitin-proteasome pathway predominantly through interaction with cullin-based E3 ligases^18^. In this scenario, USP was shown to protect Rbx1 from autoubiquitylation. CSN-associated USP15 can deubiquitinate IκBα after TNFα-mediated stimulation of the NF-κB pathway^24^. In contrary, another study found no interaction between USP15 and IκBα, and it was proposed that USP11 inhibits the ubiquitination and degradation of IκBα in the early stage while USP15 functions at a later time point in the TNFα-induced NF-κB activation^25^. USP15 also acts as a key component of the transforming growth factor β (TGF-β) signaling pathway^26^, and is a DUB for R-SMADs^27^. The DUB activity of USP15 has also been reported to be involved in the regulation of Tip110 protein degradation^28^, parkin-mediated mitochondrial ubiquitination and mitophagy^29^, and ALK3/BMPR1A in bone morphogenetic protein signaling^30^ as well as the retinoic acid-inducible gene I (RIG-I)-dependent type I IFN induction pathway^31^. Based on the insights into the characteristics of USP15 gained so far, it is conceivable that USP15 is a multifunctional protein by acting as a DUB for a wide variety of molecules in different signaling pathway. In contrast to our emerging knowledge about the ubiquitylation of HBx, little or none is known about the role of deubiquitylation for regulating HBx stability and expression levels. Here, we have identified USP15 as a critical regulator of HBx. USP15 binds to HBx and stabilizes HBx through removal of Lys^48^-linked polyubiquitin moieties from HBx, thus extending its half-life and increasing its steady-state level. The observation that treatment with the proteasome inhibitor MG132 enhanced USP15-induced HBx levels or rescued the reduction of HBx due to USP15 knockdown further supports the concept that USP15 is important for maintaining cellular pools of HBx and that upon loss of this protection HBx is targeted for proteasomal degradation. However, it should be recognized that while we observed that ectopic expression of USP15 induced a significantly higher levels of HBx than USP5, we cannot rule out the involvement of other USPs such as USP4 and USP11 which share 71 and 60% similarity at the amino acid level, respectively^32^, contributing to HBx deubiquitylation and subsequent increase of HBx levels. It is also noteworthy that in this study, HBx itself does not have any impact on USP15 protein level and its peptidase activity although the HBx protein is known capable of targeting several components of the UPS, including DDB1, the CSN, and distinct subunits of the 26 S^33^.

DUBs have been classified as oncogenes or tumor suppressors dependent on their regulatory functions on the activity of targeted proteins involved in tumor development. Since we have shown that USP15 is essential for maintaining HBx stability and that USP15 augments HBx-mediated oncogenic signals, one inference from our work is that compromising USP15 might be a novel approach to abrogate cellular transformation and serve as a target for anti-cancer therapy. Interestingly, USP15 was found to be active in various human tumor cell lines including cervical, colon, lung, brain and kidney cancers as well as lymphomas^34^. It has been recently demonstrated that inhibition of USP15 both induced tumor cell apoptosis and boosted antitumor T cell responses, and thus have important clinical applications^35^. HBx is a promiscuous transactivator that functions to regulate HBV replication, disrupt host gene expression, affect intracellular signal transduction, accelerate cell proliferation, inhibit apoptosis, and drive HCC cell migration and invasion^36^. Full length HBx is a short-lived protein *in vivo*, and many factors such as 26S proteasome complex^9^, Hsp40^10^, HBV core proteins^11^, tumor suppressor p53^12^, and transcriptional factor Id-1^13^ can decrease its protein level. Two E3 ligases, MDM2 and Siah-1, have been reported to be able to destabilize HBx^14^,^15^. Despite the ability of MDM2 to induce HBx degradation through the proteasome-dependent mechanism, MDM2 had no influence on HBx ubiquitination^14^. Conversely, Siah-1 was found to facilitate the poly-ubiquitylation modification of HBx thus predisposing it towards proteasomal degradation, and therefore attenuate its transcriptional activity^15^. Irrespective of various factors that can downregulate HBx in cells, high-level HBx is frequently observed in HCC patients and is associated with HCC progression^37^. The mechanism that sustains HBx expression in HCC at a high level is largely unknown. Our findings suggest that USP15 could protect HBx from ubiquitin-dependent proteasomal degradation and may provide a novel mechanism for the elevation of HBx that is important in the pathogenesis of HBV-related hepatocarcinoma. USP15 is a global ubiquitylation suppressor that protects many other regulatory proteins such as caspase 3, R-SMAD, TRIM25 and Nrf1 from proteasomal degradation thus affecting a wide range of signaling pathways those protein involve^27^,^32^,^38^,^39^. Moreover, while USP15 is not a transactivator protein accumulating evidence has shown that it can engage in promoter occupancy and stimulation on its own^27^,^30^. Our results obtained from the promoter reporter assays (Fig. 6) suggest that USP15 alone can stimulate various reporter genes equally well as or better than HBx. This implicates that USP15 can influence the activity of other regulatory molecules independent of HBx for transactivation function, which is further evident from the combined effect of HBx and USP15 on the promoter activation that was not even additive. Regardless, considering the ability of the viral oncoprotein HBx to affect cell functions, activate oncogenic pathways and sensitize liver cells to mutagens, it is tempting to speculate that inhibition of USP15 would interrupt chronic HBV infection, prevent the development and progression of HCC as well as foster the development of USP15-targeting strategies to expand the repertoire of molecular therapies against HCC.

## Methods

### Cell culture and transfection

Huh7, Huh7/Ct-HBx (Huh7 cells expressing a carboxyl-terminal truncated form of HBx with depletion of amino acids 130–154), HepG2 and Hep3B cells were cultured in Dulbecco’s modified Eagle’s medium (DMEM) (Invitrogen) at 37 °C in 5% CO_2_ humidified atmosphere. All mediums were supplemented with 10% fetal bovine serum (FBS), 2 μM L-glutamine (Invitrogen), 1 × MEM NEAA (Invitrogen). Huh7, Hep3B and HepG2 cells are three human HCC cell lines. Huh7 is mutated and Hep3B is deficient in p53 expression whereas HepG2 cell has wild-type p53 expression. Transient transfections were performed with X-tremeGENE HP DNA Transfection Reagent (Roche Diagnsotics) or with Lipofectamine^®^ 3000 (Invitrogen) according to the manufacturer’s instructions.

### Plasmids

pSos-HBx, pSos-HBx Δ1–25 (deletion of amino acids 1–25), pSos-HBx Δ26–50 (deletion of amino acids 26–50), pSos-HBx Δ51–80 (deletion of amino acids 51–80), pSos-HBxΔ81–120 (deletion of amino acids 81–120) and pSos-HBxΔ121–154 (deletion of amino acids 121–154) and GST-HBx expression vector pGEX-HBx have been previously constructed in our laboratory^40^. These HBx deletion mutants were made primarily based on the functional analysis of the regions of HBx important for transactivation properties^41^ with minor modifications. pHBx-FLAG expressing HBx-FLAG fusion protein and pUb-HA expressing ubiquitin-HA fusion protein were separately constructed by insertion of PCR amplified FLAG tagged HBx or HA tagged ubiquitin gene into XbaI and HindIII (New England BioLabs) sites of pcDNA3.1/myc-His(−) A (Invitrogen). The pUSP15-myc or pUSP5-myc was constructed by the insertion of a PCR-generated USP15 or USP5 gene into SalI and NotI sites of pcDNA3.1/myc-His(−) A (Invitrogen). pMyr-USP15 was constructed by the insertion of a PCR-generated USP15 gene into the SmaI and SalI sites of the plasmid pMyr (Stratagene). pCMVTNT-USP15 was generated by the insertion of a PCR-generated USP15 gene into SalI and NotI sites of pCMVTNT vector (Promega). The cis-element luciferase reporter plasmids pAP-1-luc, pAP-2-luc, pAP-3-luc, pSP-1-luc, and pNF-κB-luc were constructed as previously described^42^.

### CytoTrap yeast two-hybrid assay

Yeast two-hybrid verification was performed according to the manufacturer’s instructions. Briefly, pMyr-USP15 was co-transformed with each of the HBx constructs into temperature-sensitive mutant yeast strain cdc25Hα, which grows normally at permissive temperature (24 °C) but needs complementation by Sos protein for survival at restrictive temperature (37 °C). After replica plating, clones that grew on SD/galactose (-UL) but not on SD/glucose (-UL) plates at 37 °C were defined as “positive” which indicates the interaction of USP15 and HBx or HBx deletion mutants. Positive controls (pSosMAFB + pMyrSB and pSosMAFB + pMyrMAFB) and negative controls (pSosMAFB + pMyrLaminC and pSosColI + pMyrMAFB) were as described previously^43^.

### GST pull-down assay

*E. coli* Rosetta (DE3)(Novagen) transformed with pGEX-HBx or empty vector pGEX-4T-1 was grown and induced with 0.5 mM isopropyl-β-D-thiogalactopyranoside (IPTG). The cells were harvested and disrupted by sonication in interaction buffer (phosphate buffer saline (PBS) containing 5 mM EDTA, 1 mM DTT, 1 mM PMSF and protease inhibitor cocktail (Roche Diagnostics). After centrifuging, the supernatant was incubated with glutathione-sepharose 4B beads (GE Healthcare) and the GST immobilized beads were washed with interaction buffer. The purity and quantity of the bound GST and GST-HBx proteins were determined by examining SDS-PAGE gels stained with coomassie blue. TNT T7 Quick Coupled Transcription/Translation System (Promega) was employed to express ^35^S-labeled USP15 protein according to the manufacturer’s instructions. In brief, 2 μg of pCMVTNT-USP15 and 50 μCi of ^35^S-methionine (Amersham Biosciences) were incubated with 40 μl rabbit reticulocyte lysate for 90 min at 30 °C. For the GST-pull down assay, translated ^35^S-labeled USP15 was incubated with immobilized GST-HBx or GST beads overnight at 4 °C, and then the beads were washed five times with interaction buffer. The bound proteins were subjected to 12% SDS-PAGE. After drying the gel for 15 min, the presence of ^35^S-USP15 was detected by autoradiography.

### Western blot analysis

Cell lysates were prepared using RIPA lysis buffer (Pierce) containing a proteinase inhibitor cocktail (Roche Diagnostics). A total of 30 μg protein extracts were quantified and then subjected to electrophoresis on a 12% SDS-PAGE gel. The proteins were transferred to polyvinylidene difluoride (PVDF) membranes (Amersham Biosciences) and blocked in Tris-buffered saline (TBS) containing 2% bovine serum albumin (BSA). The specific antibodies used including anti-FLAG (Cell Signaling Technology), anti-USP15 (Santa Cruz), anti-K48-linkage Specific Polyubiquitin (Cell Signaling Technology), anti-ubiquitin (Cell Signaling Technology), anti-DDB1(Cell Signaling Technology), anti-USP5 (Santa Cruz), and anti-β-actin(Sigma Aldrich). Proteins were detected by addition of alkaline phosphatase (AP)-conjugated secondary antibody. Visualization of the immunoreactive proteins was performed by addition of CDP STAR reagents (Roche). Densitometry analysis of band signals was carried out using Image J software and the levels of HBx protein in cells transfected with pHBx-FLAG + pUSP15-myc were expressed as the relative intensity (RI) to that in the empty vector control pUSP5-myc or pHBx-FLAG after normalization to β-actin.

### Co-immunoprecipitation (Co-IP) assay

Huh7 cells were seeded on 10 cm dishes (3 × 10^6^ cells/dish), and transfected with pHBx-FLAG or empty vector pcDNA3.1/myc-His(−)A. 48 h after transfection, cells were washed three times with chilled PBS and then lysed with RIPA lysis buffer (Pierce) containing a proteinase inhibitor cocktail (Roche Diagnostics) and 1 mM PMSF. After centrifuging at 12,000 g for 10 min at 4 °C, 40 μl of EZview Red ANTI-FLAG M2 Affinity Gel (Sigma Aldrich) were incubated with the supernatant overnight. For the reverse immunoprecipitation, the supernatant was pre-cleaned with 80 μl of Protein A&G Agarose (Santa Cruz) and 0.8 μg normal mouse IgG (Santa Cruz) for 2 hours at 4 °C with gentle rotation, and then incubated with another 80 μl of Protein A&G Agarose and 1 μg USP15 antibody (Santa Cruz). After washing with cell lysis buffer for three times, the immunoprecipitated complexes were separated by 12% SDS-PAGE and analyzed by western blotting using specific antibodies as described above.

### RNA interference and siRNA transfection

40 pmol of USP15 siRNA mix (Santa Cruz) or the control siRNAs, together with the co-transfected plasmids were introduced into cells using lipofectamine 3000 Reagent (Invitrogen) following the manufacturer’s protocol. The cells were lysed with RIPA lysis buffer (Pierce) containing a proteinase inhibitor cocktail (Roche Diagnostics) and 1 mM PMSF, and analyzed by Western blot.

### Measurements of USP15 peptidase activity *in vitro*

The DUB-Glo Protease Assay (Promega) was used to measure the peptidase activity of USP15 *in vitro* following the manufacturer’s protocol. Briefly, the Z-RLRGG-Glo substrate was mixed with DUB-Glo buffer and luciferin detection reagent to form DUB-Glo reagent. The DUB-Glo^TM^ reagent was then incubated with 80 nM USP15 (Enzo Life Sciences) and increasing amount of HBx (1–80 nM) (Abcam) for 30 minutes at 22 °C. A DUB protease UCH-L3 (Boston Biochem) was used as positive control. At the end of the incubation the luminescence was measured with an Orion Microplate Luminometer (Berthold Technologies), and was recorded as relative light units (RLU). Each reaction was performed in duplicate and repeated three times.

### Determination of HBx half-life

0.75 μg of pHBx-FLAG were co-transfected into Huh7 cells with 2.25 μg of pUSP15-myc or 40 pmol of USP15 siRNA. The equal mole amounts of pcDNA3.1/myc-His(−)A or control siRNA were used as a negative control. 48 hours posttransfection, cells were treated with 100 μg/ml of cycloheximide (Cell Signaling Technology) at indicated times. Cells were washed three times with prechilled PBS and The cells were lysed with RIPA lysis buffer (Pierce) containing a proteinase inhibitor cocktail (Roche Diagnostics) and 1 mM PMSF, and followed by SDS-PAGE and Western blot analysis.

### *In vivo* ubiquitylation assay

Huh7 cells in 6-well plates were transfected with 0.4 μg pUb-HA, 0.75 μg pHBx-FLAG together with 2.14 μg pUSP15-myc or 40 pmol of USP15 siRNA. The equal mole amounts of pcDNA3.1/myc-His(−)A or control siRNA were used as a negative control. 20 h after transfection, the cells were treated with 20 μM proteasome inhibitor MG132 (Sigma Aldrich) for 6 h and then lysed with RIPA lysis buffer (Pierce) containing a proteinase inhibitor cocktail (Roche Diagnostics) and 1 mM PMSF. After incubation of the lysates with EZview Red ANTI-FLAG M2 Affinity Gel (Sigma Aldrich) for overnight at 4 °C, beads were washed with lysis buffer and the proteins were separated by 12% SDS-PAGE and analyzed by western blotting using specific antibodies including anti-K48 (1:2 000 dilution), anti-Flag (1:1 000 dilution) and anti-USP15 (1:500 dilution).

### Cis-element luciferase reporter assay

5 × 10^5^ Huh7 cells were co-transfected with 0.75 μg of pHBx-FLAG, 2.25 μg of pUSP15-myc and 0.4 μg each of pAP-1-luc, pAP-2-luc, pAP-3-luc, pSP-1-luc, pNF-κB-luc. Equal molar amounts of pcDNA3.1/myc-His(−)A were used as a negative control. 48 h after transfection, cells were lysed and 30 μg protein were used for the detection of intracellular luciferase activity (Bright-Glo Luciferase Assay System; Promega) following the manufacturer’s protocol. The light intensity was measured by a luminometer (Berthold Technologies). The relative luciferase unit (RLU) was obtained by comparison to the empty vector pCDNA3.1/myc-His(−)A and was set to ‘1’ in each experiment. Each transfection was performed in duplicate and repeated three times.

### Statistical analysis

Statistical analyses were performed with a two-tailed unpaired t test. p < 0.05 was considered statistically significant. Experiments were performed at least three times, and representative results were shown.

## Additional Information

**How to cite this article:** Su, Z.-J. *et al*. Deubiquitylation of hepatitis B virus X protein (HBx) by ubiquitin-specific peptidase 15 (USP15) increases HBx stability and its transactivation activity. *Sci. Rep.* **7**, 40246; doi: 10.1038/srep40246 (2017).

**Publisher's note:** Springer Nature remains neutral with regard to jurisdictional claims in published maps and institutional affiliations.
